# The Effectiveness of Protective Headgear in Attenuating Ball-to-Forehead Impacts in Water Polo

**DOI:** 10.3389/fspor.2019.00002

**Published:** 2019-07-10

**Authors:** Nicholas J. Cecchi, Theophil J. Oros, Derek C. Monroe, Gianna M. Fote, Wyatt X. Moscoso, James W. Hicks, David J. Reinkensmeyer

**Affiliations:** ^1^Department of Mechanical and Aerospace Engineering, University of California, Irvine, Irvine, CA, United States; ^2^Department of Ecology and Evolutionary Biology, University of California, Irvine, Irvine, CA, United States; ^3^Department of Neurology, University of California, Irvine, Irvine, CA, United States; ^4^Department of Biological Chemistry, University of California, Irvine, Irvine, CA, United States

**Keywords:** sports, concussion, head injury, head kinematics, biomechanics, head acceleration, helmet

## Abstract

Recent reports have demonstrated that there is a serious risk of head impact and injury in water polo. The use of protective headgear in contact sports is a commonly accepted strategy for reducing the risk of head injury, but there are few available protective headgears for use in water polo. Many of those that are available are banned by the sport's governing bodies due to a lack of published data supporting the effectiveness of those headgears in reducing head impact kinematics. To address this gap in knowledge, we launched a water polo ball at the forehead of an anthropomorphic testing device fitted with either a standard water polo headgear or one of two protective headgears. We selected a range of launch speeds representative of those observed across various athlete ages. Mixed-model ANOVAs revealed that, relative to standard headgear, protective headgears reduced peak linear acceleration (by 10.8–21.6%; *p* < 0.001), and peak rotational acceleration (by 24.5–48.5%; *p* < 0.001) induced by the simulated ball-to-forehead impacts. We discuss the possibility of using protective headgears in water polo to attenuate head impact kinematics.

## Introduction

Approximately 1.6–3.8 million sports-related concussions occur in the United States annually (Langlois et al., 2006). In an effort to reduce the risk of head injury, many contact sports require that athletes use protective headgears validated by scientific research to attenuate the magnitude of head impacts (Benson et al., 2009). However, the safety of athletes in some sports may be compromised by a lack of commercially available protective headgears or rules that restrict the use of protective headgears. Both of these factors contribute to the risks associated with water polo, a contact sport that presents a unique risk for head injury compared to land-based sports. Head impacts resulting from both the ball and player-to-player interactions can be frequent in water polo, with one report suggesting an average of 18.4 head impacts are sustained per game by a single team during collegiate gameplay (Cecchi et al., 2019). At the elite level, water polo has been found to carry a significant risk of head and face injury (Mountjoy et al., 2010), and a large epidemiological study found that 36% of surveyed USA Water Polo members reported sustaining at least one concussion during their playing tenure (Blumenfeld et al., 2016). Notably, 47% of athletes playing the goalie position reported sustaining a concussion and being hit in the head almost exclusively by the ball. Both goalies and defenders reported the front of the head as the most common location of impacts (Blumenfeld et al., 2016). It is difficult to suggest actionable changes to the sport based on these findings alone since the attenuation capabilities of available headgears have not been quantified.

Several kinematic measures of head movement are commonly used to quantify the magnitude of sport-related impacts and the effectiveness of protective equipment. Of these, peak linear acceleration (PLA) and peak rotational acceleration (PRA) are believed to be useful indicators of concussive injury risk (Rowson and Duma, 2013). Head injury assessment functions, such as the Gadd Severity Index and Head Injury Criterion, factor instantaneous linear acceleration and impact duration to quantify risk of more serious, life-threatening head injury (i.e., skull fracture). A pre-dominant theory is that as impact magnitude increases, injury risk also increases (Duhaime et al., 2012), but the validity of suggested “threshold” magnitudes that are capable of predicting clinical outcomes are highly contentious (Guskiewicz and Mihalik, 2011), and it is believed that concussive injury tolerance is specific to individuals (Rowson et al., 2018). However, even in the absence of concussive injury, repeated head impact exposure is suggested to pose long-term health risks that could be reduced by wearing protective headgear (McAllister and McCrea, 2017).

The use of protective headgear is a widely accepted strategy for reducing the magnitude of sports-related head impacts. There are only a few types of protective headgear available for use in water polo, and most organizations, including the National Collegiate Athletic Association (NCAA), reject their use during organized game play due to their lack of compliance with current rules and regulations (Johnson, 2014). Moreover, in contrast to other contact sports, there are currently no safety standards that water polo headgears must meet, and to the authors' knowledge there are no published data substantiating the effectiveness of water polo headgears for attenuating head impacts. The goal of this study was to compare the effectiveness of two protective headgear options in attenuating ball-to-head impacts, quantified using kinematics of the head. We hypothesize that protective water polo headgears will significantly reduce head impact kinematics relative to standard water polo headgear.

## Methods

### Experimental Setup

**Figure 1:**
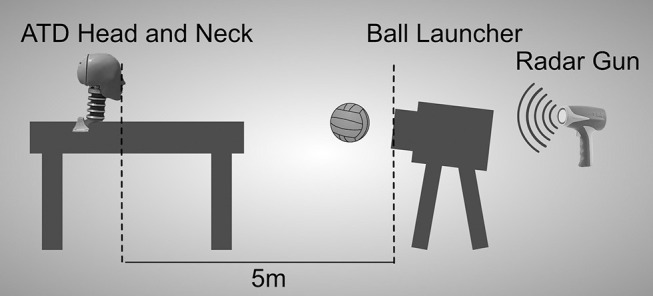
Schematic of ball launcher-anthropomorphic testing dummy (ATD) experimental setup.

In order to simulate impact kinematics of ball-to-forehead impacts in water polo, a men's size water polo ball (Kap7 International; Irvine, California) was thrown via a ball launcher at the front of an anthropomorphic testing dummy (ATD) head and neck. The ball launcher (Sidekick Soccer Machine, Seattle Sports Sciences, Inc.; Seattle, Washington) was positioned five meters from the ATD head and properly angled to send the ball at the forehead of the ATD (Figure 1). Five meters is a common shooting distance because penalty shots and shots after ordinary fouls must be taken at or behind the five-meter line (Abraldes et al., 2012). Launch speeds were calibrated and measured for each impact using a radar gun (Velocity Speed Gun, Bushnell; Overland Park, Kansas) held directly behind the ball launcher and in line with the ball's trajectory.

The ATD head and neck used were from a 50th Percentile Male Hybrid III Crash Test Dummy (Humanetics; Plymouth, Michigan). The head and neck apparatus were securely mounted to a heavy table that was immovable by the ball-to-forehead impacts. DTS Sliceware software (Diversified Technical Systems, Inc.; Seal Beach, California) was used to calibrate, arm, and record impacts from the ATD. A DTS SLICE Nano in-dummy data acquisition system comprised of BASE and BRIDGE modules was used to record three channels of linear acceleration and three channels of rotational velocity in the X, Y, and Z directions from MSI Model 64C-2000 accelerometers and DTS ARS PRO-8k angular rate sensors mounted inside of the ATD at its center of gravity.

### Testing Procedure

The water polo ball was launched at four speeds (11.2, 15.6, 20.1, and 24.6 m/s) that encompassed a range of shot speeds observed in water polo-related research (Whiting et al., 1985; Abraldes et al., 2012; Uljevic et al., 2013). Three headgears were worn by the ATD. The first headgear worn was a standard water polo cap (Turbo; Barcelona, Spain). Standard water polo caps consist of one thin layer of a polyester-PBT blend fabric and two ear guards on the sides of the head. The second headgear worn was the Kap7 Head Guard (Kap7 International; Irvine, California). The Head Guard is a thick silicone cap that features padded dimples across its entire outer surface; the padded dimples absorb some of the energy from head impacts. The Head Guard is designed to be worn underneath a standard water polo cap and was used according to manufacturer instructions in this experiment. The third headgear tested was a prototype of the Counter Tuff-Cap (Counter, Inc.; Anaheim, California). The Tuff-Cap has a similar appearance to a standard water polo cap, but features a thin layer of Poron XRD (Rogers Corporation; Chandler, Arizona) sandwiched between two layers of polyester-PBT fabric. Poron XRD is a rate-dependent smart material—soft and flexible at rest, but dissipates forces upon impact—commonly found in other sports helmets and protective equipment. All headgears were soaked in chlorinated pool water until they were fully saturated prior to each impact trial in order to best replicate how the headgears would be used during gameplay. Nine impact trials were recorded at each speed-headgear combination.

Before each ball launch, the ATD was calibrated and armed. After each impact, the software immediately downloaded a region of interest 100 ms pre-impact and 100 ms post-impact at a sampling rate of 20,000 Hz. The launch speed was recorded for each impact and only trials that were within ±0.9 m/s of the target launch speed were counted and recorded for analysis. Successful impact trials were determined by visual confirmation of impact with the capped portion of the ATD forehead and proper post-impact ball trajectory. An accurate launch to the forehead of the ATD sent the ball upwards after impact. If the shot missed the forehead, such as with a complete miss, skim, or facial impact, it was recognized by an aberrant post-impact trajectory of the ball. In order to standardize impact location (on the head) for all trials and minimize the confounding effects of ball spin, impacts that did not yield proper post-impact ball trajectory were discarded and repeated.

### Data Processing

Resultant linear accelerations and rotational velocities were computed from the ATD data filtered with SAE J211 compliant CFC 1000 and CFC 180 filters, respectively. Rotational acceleration values were obtained by differentiating the rotational velocity data using a custom script in MALTAB 2017a (MathWorks; Natick, MA). PLA, expressed in g (1 g ≈ 9.81 m/s^2^), and PRA, expressed in krad/s^2^, were extracted for analysis.

### Statistical Analysis

Separate speed (11.2, 15.6, 20.1, 24.6 m/s) by headgear (standard cap, Kap7 Head Guard, Counter Tuff-Cap) mixed-model analysis of variance (ANOVAs), with speed as the repeated measure, were used to test for differences in PRA and PLA. Corrections for sphericity (Huynh-Feldt epsilon, ε) and partial eta-squared (η^2^) effect sizes are reported where necessary. Simple main effects of headgear were decomposed with a series of *post-hoc t*-tests with Bonferroni corrections for multiple comparisons (three comparisons; *p* < 0.016). Corrected *p*-values and Cohen's *d* effect sizes are reported.

## Results

There were simple main effects of headgear [*F*_(2, 24)_ = 34.311, *p* < 0.001, η^2^ = 0.741] and speed [*F*_(3, 72)_ = 44.665, *p* < 0.001, ε = 1.000, η^2^ = 0.650] on PRA. The Head Guard (*p* < 0.001, *d* = 2.59) and Tuff-Cap (*p* < 0.001, *d* = 3.26) reduced PRA relative to standard headgear. There were simple main effects of headgear [*F*_(2, 24)_ = 26.694, *p* < 0.001, η^2^ = 0.690] and speed [*F*_(2.791, 66.985)_ = 241.645, *p* < 0.001, ε = 0.930, η^2^ = 0.910] on PLA. The Head Guard (*p* < 0.001, *d* = 3.36) and Tuff-Cap (*p* < 0.001, *d* = 3.39) reduced PLA relative to standard headgear. No differences in PLA or PRA were observed between the Head Guard and Tuff-Cap (*p* > 0.507) (Table 1).

**Table 1:** Headgear testing results during ball-to-forehead impacts.

**Ball speed (m/s)**	**Headgear**	**Mean outcome measures (SD)**	
		**Peak linear acceleration (g)**	**Peak rotational acceleration (krad/s^**2**^)**
	Standard cap	64.0 (6.7)	5.5 (1.0)
24.6	Kap7 Head Guard	57.1 (6.9)	4.1 (0.9)^**^
	Counter Tuff-Cap	51.5 (7.0)^**^	4.1 (0.6)^**^
	Standard cap	48.1 (4.0)	4.9 (1.3)
20.1	Kap7 Head Guard	42.6 (5.5)	3.7 (0.9)
	Counter Tuff-Cap	42.0 (4.1)^*^	3.7 (0.5)^*^
	Standard cap	37.0 (2.6)	3.6 (1.0)
15.6	Kap7 Head Guard	32.3 (2.8)^*^	2.3 (0.6)^**^
	Counter Tuff-Cap	30.6 (4.4)^**^	2.3 (0.3)^**^
	Standard cap	26.9 (4.2)	3.3 (1.3)
11.2	Kap7 Head Guard	21.1 (3.3)^**^	1.8 (0.4)^***^
	Counter Tuff-Cap	23.1 (2.1)	1.7 (0.3)^***^

Asterisks denote differences between protective headgear and standard cap. (

* *corrected p < 0.05*,

** *corrected p < 0.01*,

*** *corrected p < 0.001)*.

## Discussion

Substantial research efforts have been expended to investigate the protective ability of various padded sports headgears, and the results of these studies have shown that certain padded headgears are capable of attenuating head impacts (Broglio et al., 2003; Naunheim et al., 2003a; McIntosh and Patton, 2015). To the authors' knowledge, this is the first investigation to quantify the protective capabilities of protective water polo headgears. Despite water polo being an aggressive sport that is associated with a high risk of head impact and injury, standard headgear that is currently used across competitive levels of the sport lacks features that aim to attenuate head kinematics, and regulations set by many of the sport's governing bodies prohibit the use of padded protective headgear.

We selected ball launch speeds that represent a range of water polo shot speeds commonly observed across various age levels, from youth to elite adult athletes. Soccer is another sport in which athletes regularly sustain ball-to-forehead impacts, and the impact magnitudes (PLA, PRA) we report are similar to those observed in soccer players during gameplay (Caccese et al., 2016) and in experimental reconstruction using machine-launched soccer balls at similar speeds (Naunheim et al., 2003b; Wirsching et al., 2019). Increases in blood-based biomarkers of brain injury have been related to ball-to-head impacts sustained during soccer competitions (Stålnacke et al., 2004, 2006) and during practice, from machine-launched balls (Wallace et al., 2018; Wirsching et al., 2019). Similar comparisons of neurological and physiological outcomes in water polo players, with and without protective headgear during gameplay, are warranted.

Recent changes to NCAA rules have banned padded headgears that are worn beneath water polo caps (i.e., Kap7 Head Guard) (Johnson, 2014). Soft headgears are currently banned by the NCAA for being ineffective at reducing the rotational kinematics of head impacts and therefore, the risk of concussion (NCAA Committee on Competitive Safeguards and Medical Aspects of Sports, 2013). The two protective headgears we tested here significantly reduced PRA of head impacts across most speeds relative to standard headgear. Whether reductions of PRA by an average of 1.3 krad/s^2^ are clinically meaningful for injury prevention warrants further investigation, and protective standards similar to those for other contact sports should be implemented to determine the effectiveness of water polo headgears. However, the data presented here suggest that the use of protective headgears, especially at the elite competition level where athletes are exposed to the highest ball speeds, may reduce cumulative head impact exposure sustained by water polo athletes and mitigate the risk for head injury.

A key limitation of our study is that we only recorded impacts to the front of the head. Survey data indicate that this is the most common area for goalies and defenders to be impacted (Blumenfeld et al., 2016); however, previous work with other protective headgears has demonstrated that impact attenuation is location dependent for different headgears in other sports (O'Sullivan et al., 2013; Nur et al., 2015; O'Sullivan and Fife, 2016). Future comparisons of protective headgears should test multiple sites encompassing the entirety of the head. We also only studied the effects of wearing protective headgear during ball-to-forehead impacts, though player-to-player interactions are known to result in head contact between field players. Notwithstanding these limitations, we report the first data demonstrating effectiveness of protective headgear for water polo athletes at reducing the kinematic effects of simulated ball-to-head impacts.

This study compared two protective water polo headgears and their ability to attenuate head kinematics, relative to a standard water polo cap, resulting from ball-to-forehead impacts. The results demonstrate that, despite rules and regulations banning some headgears during gameplay, the protective water polo headgears tested here are able to significantly reduce kinematic measures associated with head injury. This suggests that teams or individuals may benefit from wearing protective water polo headgears during practices. Further evidence of headgear efficacy would encourage governing bodies to adjust rules and regulations that currently prohibit their use during sanctioned gameplay.

## Data Availability

The datasets generated for this study are available on request to the corresponding author.

## Author Contributions

NC, TO, WM, JH, and DR contributed to the conception and design of the study. NC, TO, and GF collected and pre-processed data. DM performed the statistical analysis. NC and DM wrote the first draft of the manuscript. TO and GF wrote sections of the manuscript. All authors contributed to manuscript revision, read, and approved the submitted version.

### Conflict of Interest Statement

NC and TO have a financial interest in Counter, Inc. The remaining authors declare that the research was conducted in the absence of any commercial or financial relationships that could be construed as a potential conflict of interest.
